# Abnormal topological parameters in the default mode network in patients with impaired cognition undergoing maintenance hemodialysis

**DOI:** 10.3389/fneur.2022.951302

**Published:** 2022-08-18

**Authors:** Chuanlong Cao, Die Zhang, Wanqing Liu

**Affiliations:** ^1^Department of Radiology, West China Second University Hospital, Sichuan University, Chengdu, China; ^2^Department of Radiology, Affiliated Xinhua Hospital of Dalian University, Dalian, China; ^3^Department of Radiology, National Clinical Research Center for Infectious Disease, Shenzhen Third People's Hospital, The Second Affiliated Hospital, School of Medicine Southern University of Science and Technology, Shenzhen, China; ^4^Key Laboratory of Birth Defects and Related Diseases of Women and Children (Sichuan University), Ministry of Education, Chengdu, China

**Keywords:** end-stage renal disease, default mode network, graph theory, functional connectivity, resting-state fMRI

## Abstract

**Objective:**

The role of the default mode network (DMN) in the cognitive impairment experienced by patients with end-stage renal disease (ESRD) undergoing maintenance hemodialysis (MHD) remains unknown. This study tested the hypothesis that the topological architecture of the DMN plays a key role in ESRD-related cognitive impairment.

**Methods:**

For this study, 43 ERSD patients receiving MHD and 41 healthy control (HC) volunteers matched for gender, age and education underwent resting-state functional magnetic resonance imaging examinations. DMN architecture was depicted by 20 selected DMN subregions. Graph theory approaches were applied to investigate multiple topological parameters within the DMN in resting state at the global, local and edge levels.

**Results:**

Globally, the MHD group exhibited topological irregularities as indicated by reduced values for the clustering coeffcient (C_p_), normalized C_p_ (γ), world-index (σ), and local effciency (E_loc_) compared with the HC group. Locally, the MHD group showed greater nodal betweenness in the left retrosplenial cortex (RC) compared with the HC group. At the edge level, the MHD group exhibited disconnected resting-state functional connections (RSFCs) in the medial temporal lobe (MTL) subsystem including the ventral medial prefrontal cortex (VMPC)–left posterior inferior parietal lobule, VMPC–right parahippocampal cortex (PC), and right RC–left PC RSFCs. Additionally, the VMPC–right PC RSFC was positively correlated with the Digit Span Test score and E_loc_, and the right RC–left PC RSFC was positively correlated with the Montreal Cognitive Assessment score and E_loc_ in the MHD group.

**Conclusions:**

ESRD patients undergoing MHD showed local inefficiency, abnormal nodal centralities, and hypoconnectivity within the DMN, implying that the functional differentiation and local information transmission efficiency of the DMN are disturbed in ESRD. The disconnected RSFCs in the MTL subsystem likely facilitated topological reconfiguration in the DMN of ESRD patients, leading to impairments of multidomain neurocognition including memory and emotion regulation.

## Introduction

End-stage renal disease (ESRD) is characterized by metabolic disorder and multiple organ dysfunction and usually involves a glomerular filtration rate <15 ml/(min. 1.73 m^2^) (1). Maintenance hemodialysis (MHD) is one of the most important and common treatment methods for patients with ESRD (2). Unfortunately, studies have found that 30–60% of ESRD patients undergoing MHD experience cognitive impairment (3), including executive function impairments, memory disorders and emotional disturbance (4–6). Notably, even young ESRD patients exhibit worse cognitive function than their healthy peers (7). However, the neurological basis of this neurocognitive decline in ESRD patients undergoing MHD is unclear. Therefore, efforts to identify potential imaging biomarkers for impaired cognition in ESRD patients undergoing MHD are vital to the early clinical detection of such impairment, application of timely intervention, and improvement of prognosis for ESRD patients.

Recently, neuroimaging technology has been widely used to study the neuropathological mechanism of cognition in patients with ESRD. Structural magnetic resonance imaging (MRI) studies in ESRD patients have revealed atrophy of gray matter in the posterior cingulate cortex and precuneus cortex (8–10) and diminished white matter integrity in frontal and temporal regions (11, 12). Moreover, resting-state functional magnetic resonance imaging (rs-fMRI) in ESRD patients has shown abnormalities in the regional homology (13) and amplitude of low-frequency fluctuation (14) as well as fragmented functional connections (FCs) (15–17) in the posterior cingulate cortex, precuneus cortex, and medial temporal lobe (MTL). The neuroanatomical areas mentioned above are core sections of the default mode network (DMN). However, the previous studies were limited to assessment of the spontaneous abnormalities in local DMN subregions and did not directly assess the architecture of the whole-DMN functional networks in patients with ESRD.

According to a previous study (18), the DMN can be divided into three functionally independent subsystems: (1) the cortical hub subsystem consisting of the posterior cingulate cortex (PCC)and the anterior medial prefrontal cortex (aMPFC); (2) the dorsal medial prefrontal cortex (dMPFC) subsystem consisting of the dMPFC, temporal parietal junction (TPJ), lateral temporal cortex (LTC), and temporal pole (TP); and (3) the MTL subsystem consisting of the ventral MPFC (VMPC), posterior inferior parietal lobule (PIPL), retrosplenial cortex (RC), parahippocampal cortex (PC), and hippocampal formation (HF) (19, 20). The main functions of the DMN are to maintain the most fundamental cognitive activities of the human brain in the resting state, to perceive external information, to monitor the mental state of self-behavior, and to participate in cognitive activities (18). Previous behavioral studies have shown that patients with ESRD have multidomain DMN-related neurocognitive impairment (21), which may represent the potential neuropathic mechanism of cognitive decline in ESRD. Nevertheless, the functional state of the DMN in ESRD has yet to be characterized, even though the DMN participants in vital cognitive processes.

Graph theory is a widely applied approach to analyzing the topology of the brain connectome, as it can reveal the internal working mechanism of the network at the whole-brain network level (22). Graph theory analysis of the DMN has been performed for many cognitive decline-related diseases and can provide key information regarding the potential mechanism by which the DMN topology contributes to disease (23, 24). Functional changes in the DMN can be identified *via* quantitative MRI evaluation, and such findings may contribute to improvements in clinical prognoses and diagnoses (25). A series of rs-fMRI studies related to ESRD that used graph theory at the whole network level has been published (26–29). Although a whole-brain graph theory analysis could reveal the changes in the global and local topological parameters of the whole-brain of ESRD patients, this approach does not allow observation of the changes in the topological parameters of the DMN (especially the global parameters such as the small-world parameters), which was shown to be damaged in ESRD patients. Hence, the topological alterations of the DMN at the subnetwork level in ESRD patients undergoing MHD need to be further studied.

In the present study, we hypothesized that ESRD-based cognitive impairment might be related to changes in the topological pattern of the DMN. To test this hypothesis, we applied graph theory analysis based on rs-fMRI data to explore the topology of the DMN in ESRD patients undergoing MHD at the global, local, and edge levels and assessed the relationships between neuronal parameters and clinical parameters.

## Materials and methods

### Participants

For this study, 43 patients with ESRD who were receiving maintenance hemodialysis (MHD group) were recruited from the hemodialysis room of the Affiliated Zhongshan Hospital to Dalian University from October 2016 to December 2018. In the same period, 41 healthy control (HC) volunteers matched with the MHD group for gender, age and years of education were recruited in the local community. The study protocol was approved by the hospital ethics committee, and all subjects voluntarily participated and signed an informed consent form.

The inclusion criteria included: (1) right handedness; (2) age 25–70 years; (3) regular dialysis for at least the previous 3 months in the MHD group; and (4) a Montreal Cognitive Assessment (MoCA) score of ≥26 points in the HC group. The exclusion criteria included: (1) contraindications for MRI, such as claustrophobia and metal foreign bodies in the body; (2) drug addiction, alcohol addiction, etc.; (3) inability to complete the test due to visual impairment or limb dysfunction; (4) mental and relevant nervous system diseases, such as severe brain injury, obvious lesions in the brain, epilepsy, schizophrenia, etc.; (5) history of diabetic nephropathy or hypertensive nephropathy; and (6) MRI image post-processing head movement >2.0°.

### Neuropsychological tests

Overall cognition was evaluated in all participants by administering the Chinese version of the MoCA. Then participants executive functioning and short-term and delayed memory were tested using the Trail Making Test A (TMT_A) and Digit Span Test (DST). Participants also completed the Zung's Self-Rating Anxiety Scale (SAS) and Zung's Self-Rating Depression Scale (SDS) for evaluation of anxiety and depression, respectively. To reduce external interference, the tests were carried out in a quiet and independent environment. First, any concerns of the participant were addressed in brief conversation, and then the testing began once the participant was relaxed and adapted to the environment. All neuropsychological scale tests were performed within 1 h after MRI scanning and within a total time of ~25–30 min.

### Laboratory examinations

For patients in the MHD group, blood samples were collected and biochemical analysis was performed within 24 h before MRI scanning. The tested values included: (1) renal function indexes: urea and creatinine; and (2) routine blood tests and electrolyte levels: red blood cell (RBC) count and K^+^, Ca^2+^, and P^3+^ levels. These laboratory data were not collected for the HC group.

### MRI data acquisition

All study participants underwent MRI examination performed by a Siemens 3.0T MR scanner with a standard 12-channel head coil in the Department of Radiology, Affiliated Zhongshan Hospital to Dalian University. The scan sequence included: (1) T2-weighted-fluid-attenuated inversion recovery (T2-FLAIR) scan: repetition time (TR) = 4,000 ms, echo time (TE) = 77 ms, field of view (FOV) = 250 × 226 mm, matrix (MS) = 256 × 180, flip angle (FA) = 150°, number of layers (slice) = 20 and layer thickness (ST) = 5.0 mm; (2) three-dimensional T1-weighted imaging (3D-T1WI) scan: TR =2,530 ms, TE = 2.22 ms, FOV = 224 × 224 mm, MS = 224 × 224, FA = 7°, slice = 192, ST = 1 mm; and (3) rs-fMRI scan with single excitation imaging gradient echo sequence: TR = 2,000 ms, TE = 30 ms, FOV = 224 × 224 mm, MS = 64 × 64, FA = 90°, slice = 31, ST = 3.5 mm, time points = 240.

During resting state data collection, all participants were asked to lie quietly on the examination bed, and the head was fixed with foam sponge to reduce the impact of head movement. They also were instructed to remain quiet with eyes closed and to try to avoid unnecessary head movement and specific thinking activities. After the examination, all participants were asked briefly to report whether they fell asleep during MRI data acquisition.

### Image pre-processing

The Data Processing Assistant for Resting-State fMRI (DPARSF) (30) software package (http://rfmri.org/DPARSF) based on the MATLAB 2013b platform (Mathworks, Natick, MA, USA) was used for preprocessing of images. The specific steps were as follows: (1) removal of the first 10 time points to eliminate the potential noise impact of an uneven magnetic field at the beginning of scanning; (2) slice timing: elimination of the time phase difference in interval scanning; (3) realignment: six parameter rigid body linear transformation was used to align the participant's functional image to the first time point; (4) spatial normalization: the individual T1WI was aligned to the average functional image through six degrees of freedom linear transformation, and then the structural image was divided into gray matter, white matter, and cerebrospinal fluid. Finally, based on the segmentation results, the functional image for the participant was transformed from individual space to MNI space using the Diffeomorphic Anatomical Registration Through Exponentiated Lie Algebra (DARTEL) tool (31); (5) spatial smoothing: application of 4-mm-full-width at half- maximum (FWHM) Gaussian kernel; (6) further linear detrending of the time series; (7) noise regression: the Friston24 parameter model (32, 33) was used to eliminate the head motion effect on the aligned functional image. In addition, white matter and cerebrospinal fluid signals were regressed to diminish the effects of respiration and heartbeat; and (8) temporal filtering: the frequency band (0.01–0.1 Hz) was selected for filtering to remove the effect of physiological high-frequency noise and low-frequency drifts. According to an established standard (34), participants were excluded for head motion corresponding to a frame-wise displacement (FD) >2 mm.

### Construction of the DMN connectome

In graph theory, a network consists of a series of nodes and edges. According to the study by Andrew-Hanna et al. (18), we selected 20 specific DMN subregions as regions of interest (ROIs) and defined them as nodes (Table 1). The average time series for each ROI voxel was extracted using 6-mm radius spherical seeds based on the MNI coordinate system, and the Pearson correlation coefficient for each ROI–ROI pair was calculated as the edge representing ROI–ROI resting-state FC (RSFC). Fisher's r-to-z transformation was performed to make the results better align with a normal distribution. The Graph Analysis Toolbox (GAT) (35) was employed for analysis of the topology of the DMN.

**Table 1:** ROI of DMN.

**ROI of DMN**	**Abbreviation**	**BA**	**MNI**		
			**x**	**y**	**z**
**Core subsystem**					
Anterior medial prefrontal cortex	aMPFC.R	10, 32	6	52	−2
	aMPFC.L		−6	52	−2
Posterior cingulate cortex	PCC.R	23, 31	8	−56	26
	PCC.L		−8	−56	26
**DMPFC subsystem**					
Dorsal medial prefrontal cortex	dMPFC	9, 32	0	52	26
Temporal parietal junction	TPJ.R	40, 39	54	−54	28
	TPJ.L		−54	−54	28
Lateral temporal cortex	LTC.R	21, 22	60	−24	−18
	LTC.L		−60	−24	−18
Temporal pole	TP.R	21	50	14	−40
	TP.L		−50	14	−40
**MTL subsystem**					
Ventral medial prefrontal cortex	VMPC	11, 24, 25, 32	0	26	−18
Posterior inferior parietal lobule	PIPL.R	39	44	−74	32
	PIPL.L		−44	−74	32
Retrosplenial cortex	RC.R	29, 30, 19	14	−52	8
	RC.L		−14	−52	8
Parahippocampal cortex	PC.R	20, 36, 19	28	−40	−12
	PC.L		−28	−40	−12
Hippocampal formation	HF.R	20, 36	22	−20	−26
	HF.L		−22	−20	−26

MNI, Montreal Neurological Institute; BA, Brodmann areas. Each node extracts the average time series of each ROI with spherical seeds with a radius of 6 mm.

### Sparsity range selection

To ensure the network had the same number of edges and to minimize the impact of possible differences in the overall correlation strength between groups, subsequent comparisons between networks were performed using the threshold method for network sparsity (S), which is defined as the fraction of the number of existing edges divided by the maximum possible number of edges in a network. In the present study, the network sparsity range was 0.10 ≤ S ≤ 0.34 with a step size of 0.01, which allowed for small-world topological properties to be properly estimated (36).

### Network topology metrics

Descriptions of the analyzed network topology metrics have been interpreted in Supplementary material, and concise summaries of the network parameters have been provided in our previous studies (37, 38). Node global topology metrics included the clustering coeffcient (Cp), shortest path length (Lp), normalized clustering coefficient (γ), normalized shortest path length (λ), small-world index (σ), global effciency (E_glob_), and local effciency (E_loc_). Additionally, node local topology metrics included nodal betweenness and nodal degree. Furthermore, the network edge in our study was defined as a ROI–ROI RSFC.

### Statistical analysis

The data for demographic statistical parameters, neuropsychological scale scores and laboratory indicators all conformed to a normal distribution. Differences in demographic and clinical data between the MHD and HC groups were identified using a two-sample *t*-test with the IBM SPSS statistics 20.0 software (IBM, Armonk, NY, USA). Differences in global and regional network parameters between the two groups were detected using two-tailed non-parametric permutation tests (implemented in GAT) with 1,000 repetitions. To eliminate the influence of the sparsity threshold, we performed area under the curve (AUC) analysis to further detect differences in network metrics between the MHD and HC groups. False discovery rate (FDR) correction was applied as a multiple comparison correction in the regional topology analysis, with a FDR *q* < 0.05 indicating statistical significance (39). Differences in the network edges within the DMN between the two groups were identified using a two-sample *t*-test with the Network-Based Statistic (NBS) approach (5,000 permutations) corrected for multiple comparisons (40). The relationships among altered global and regional network metrics, abnormal RSFCs, and neuropsychological data (MoCA, TMT- A, DST, SAS, and SDS) in the MHD group were investigated using Pearson correlation analysis. Meanwhile, age, gender, education, and head motion were used as covariates in graph analysis. Values of *p* < 0.05 indicated statistically significant differences between the groups.

## Results

### Demographic and clinical data of study participants

As shown in Table 2, no differences in age, gender, education, or head motion were observed between the MHD and HC groups (all *p* > 0.05). The MHD group had lower MoCA and DST scores and higher TMT-A, SAS, and SDS scores compared with the HC group (all *p* < 0.05).

**Table 2:** Demographic and clinical information of MHD and HC.

**Characteristics**	**MHD**	**HC**	***t*-value**	***p*-value**
	**(*n* = 43)**	**(*n* = 41)**		
Age (years)	49.70 ± 10.50	51.46 ± 10.68	−0.764	0.447
Gender (male/female)	31/12	27/14	–	0.536
Education (years)	11.02 ± 2.76	11.88 ± 2.98	−1.363	0.587
Head motion (mean FD, mm)	0.13 ± 0.05	0.11 ± 0.05	1.555	0.124
MoCA (score)	25.53 ± 2.36	27.41 ± 1.40	−4.462	<0.001^*^
TMT-A (sec)	67.27 ± 27.28	47.36 ± 16.35	4.079	<0.001^*^
DST (score)	11.88 ± 2.42	13.41 ± 2.96	−2.601	0.011^*^
SAS (score)	38.58 ± 9.08	30.58 ± 4.33	5.076	<0.001^*^
SDS (score)	44.02 ± 11.27	32.12 ± 7.01	5.648	<0.001^*^
**Laboratory data**				
Creatinine (μmol/L)	427.17 ± 205.45	–	–	–
Urea (mmol/L)	10.18 ± 4.21	–	–	–
K^+^ (mmol/L)	3.57 ± 0.63	–	–	–
Ca^2+^ (mmol/L)	1.89 ± 0.29	–	–	–
P^3+^ (mmol/L)	1.02 ± 0.52	–	–	–
RBC (10^12^/L)	3.46 ± 0.64	–	–	–

Data are presented as the mean ± SD.

* p < 0.05 was considered statistically significant.

FD, frame-wise displacement; MoCA, Montreal cognitive assessment; TMT- A, Trail Making Test A; DST, Digit span test; SAS, Zung's self-rating anxiety scale; SDS, Zung's self-rating depression scale; RBC, red blood cell.

### Altered node topology metrics within the DMN

**Figure 1:**
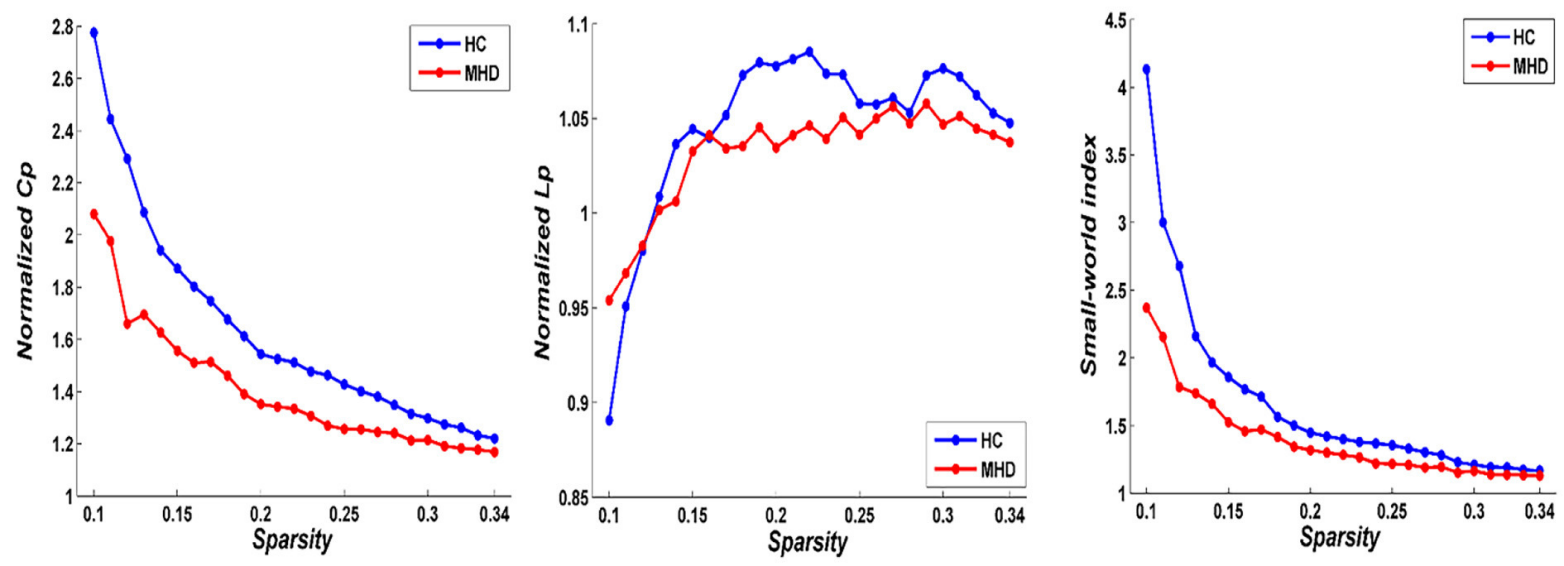
Small-world topological properties of DMN in MHD group (*n* = 43) and HC group (*n* = 41). In the network sparsity from 0.10 to 0.34. The Normalized Cp (γ) was obviously larger than 1, the Normalized Lp was approximately equal to 1, and the Small-world index (σ) larger than 1 in the DMN of the two groups, suggesting that both MHD and HC groups presents typical features of small-world topological properties.

**Figure 2:**
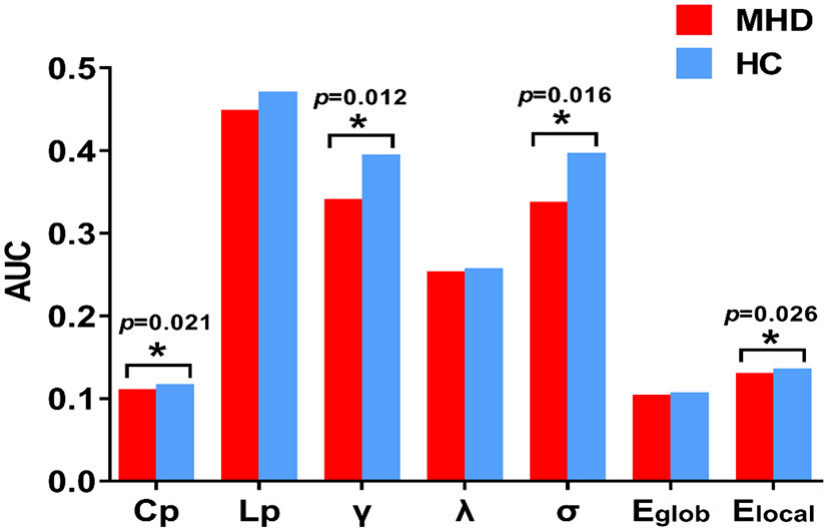
Bar graph showing the average AUC value of global network metrics in MHD (red) and HC (blue) group in the network sparsity from 0.10 to 0.34. The AUC values of the C_p_, γ, σ, and E_loal_ in MHD group was significantly lower compared with the HC group (*p < 0.05, uncorrected). AUC, area under the curve; Cp, clustering coefficient; Lp, shortest path length; γ, normalized clustering coefficient; λ, normalized shortest path length; σ, small-world-ness; E_glob_, global efficiency; E_loc_, local efficiency; MHD, maintenance hemodialysis; HC, healthy control.

**Figure 3:**
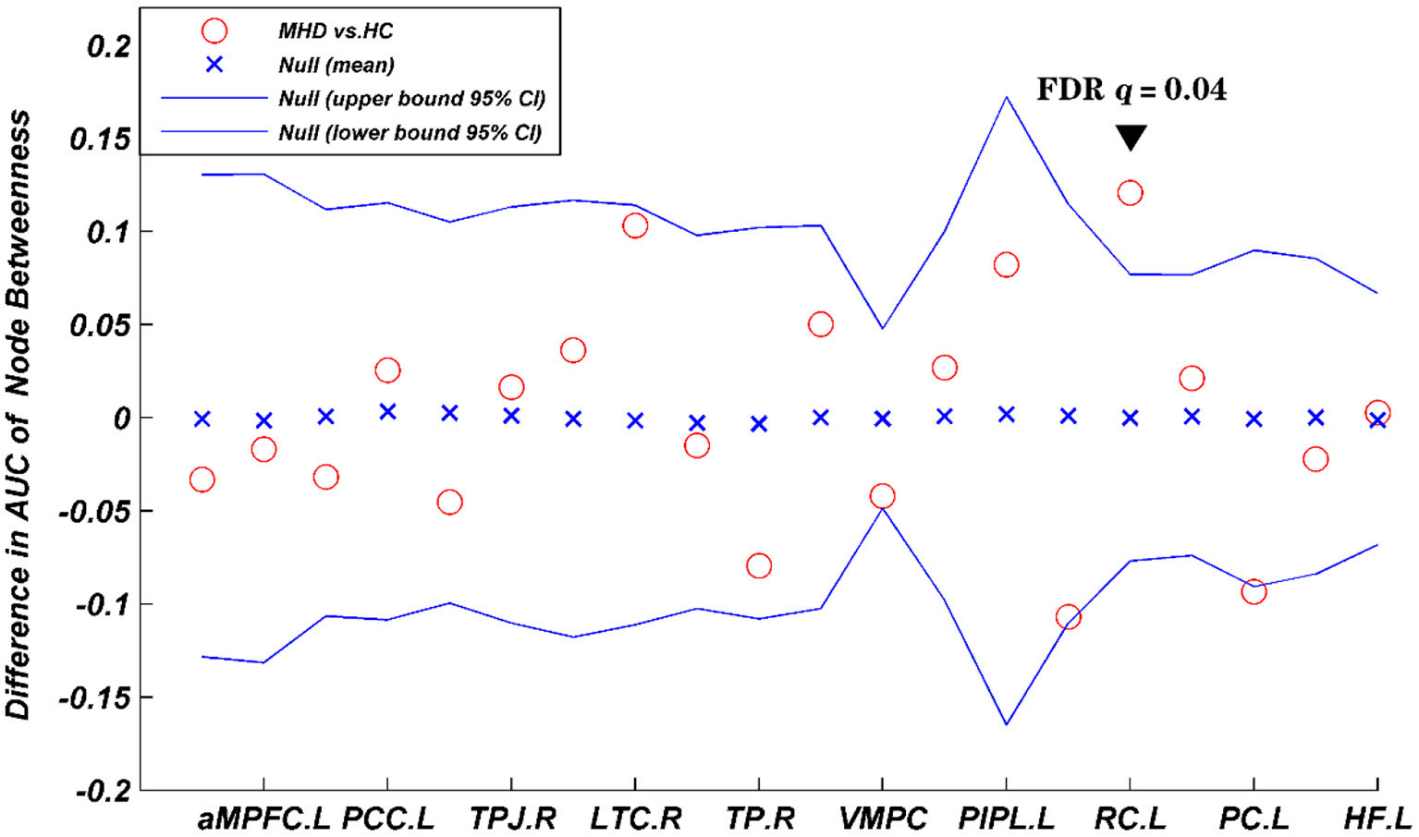
Inter-group differences in the node betweenness of the MHD group (*n* = 43) and HC group (*n* = 41) in the network sparsity from 0.10 to 0.34. The 95% confidence interval and inter-group difference of the node betweenness. The red circles indicate differences between the two groups; The back triangle indicate significant inter-group differences in left RC (FDR *q* = 0.04). Positive values indicate MHD > HC, negative values indicate MHD < HC. L, left; R, right; PCC, posterior cingulate cortex; aMPFC: anterior medial prefrontal cortex; dMPFC, dorsal medial prefrontal cortex; TPJ, temporal parietal junction; LTC, lateral temporal cortex; TP, temporal pole; VMPC, ventral medial prefrontal cortex; PIPL, posterior inferior parietal lobule; RC, retrosplenial cortex; PC, parahippocampal cortex; HF, hippocampal formation.

In both the MHD and HC groups, the DMN exhibited characteristics of small-world topology (σ > 1, γ > 1, and λ ≈ 1) in the network sparsity range from 0.10 to 0.34 (Figure 1), which could promote brain functional differentiation and integration as well as information processing, while maximizing efficiency and decreasing energy consumption to achieve the optimal balance between information separation and integration. However, the AUC values for the C_p_ (*p* = 0.021), γ (*p* = 0.012), σ (*p* = 0.016), and E_loal_ (*p* = 0.026) within the DMN in the MHD group were significantly lower than those for the HC group in the network sparsity range from 0.10 to 0.34 (*p* < 0.05, uncorrected). There were no significant differences in the L_p_ (*p* = 0.058), λ (*p* = 0.290), and E_glob_ (*p* = 0.204) within the DMN between the two groups in the same sparsity range (Figure 2). Locally, the MHD group showed greater nodal betweenness in the left RC (FDR *q* = 0.04) compared with the HC group (Figure 3).

### Altered network edges within the DMN

**Figure 4:**
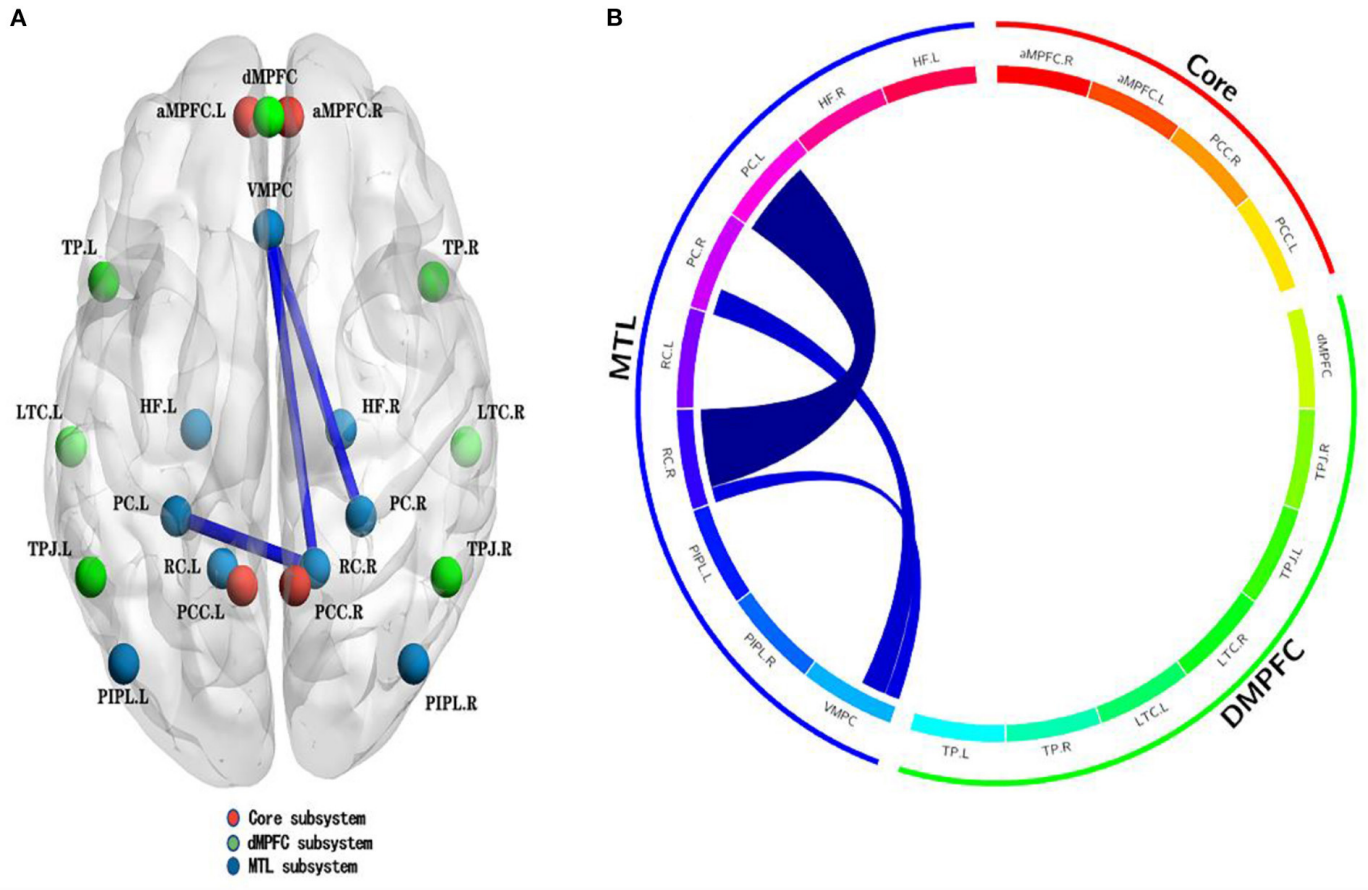
Abnormal RSFCs within DMN between the MHD group (*n* = 43) and HC group (*n* = 41). The blue edges represent decreased RSFCs within the DMN. These decreased edges are located in the medial temporal lobe (MTL) subsystem within the DMN **(A,B)**. Undirected edges correspond to *t*-values, with a larger *t*-value corresponding to a thicker edge (*P* < 0.05, NBS corrected). L, left; R, right; PCC, posterior cingulate cortex; aMPFC: anterior medial prefrontal cortex; dMPFC, dorsal medial prefrontal cortex; TPJ, temporal parietal junction; LTC, lateral temporal cortex; TP, temporal pole; VMPC, ventral medial prefrontal cortex; PIPL, posterior inferior parietal lobule; RC, retrosplenial cortex; PC, parahippocampal cortex; HF, hippocampal formation.

The MHD group exhibited weaker RSFCs of the VMPC–left PIPL, VMPC–right PC and right RC–left PC located in the MTL subsystem within the DMN compared with the HC group (*p*_NBS_ < 0.05; Table 3, Figure 4).

**Table 3:** Abnormal RSFCs within DMN between MHD and HC.

**DMN subregion**	**DMN subregion**	***t*-value**	***p*-value**
VMPC	PIPL.L	−3.520	<0.001^#^
VMPC	PC.R	−3.618	<0.001^#^
RC.R	PC.L	−4.072	<0.001^#^

Abnormal RSFCs within the DMN between MHD and HC (p < 0.05, # represents NBS correction). L, left; R, right; VMPC, Ventral medial prefrontal corte; PIPL, posterior inferior parietal lobule; PC, parahippocampal cortex; RC, retrosplenial cortex.

### Correlations between network measures and clinical data

**Figure 5:**
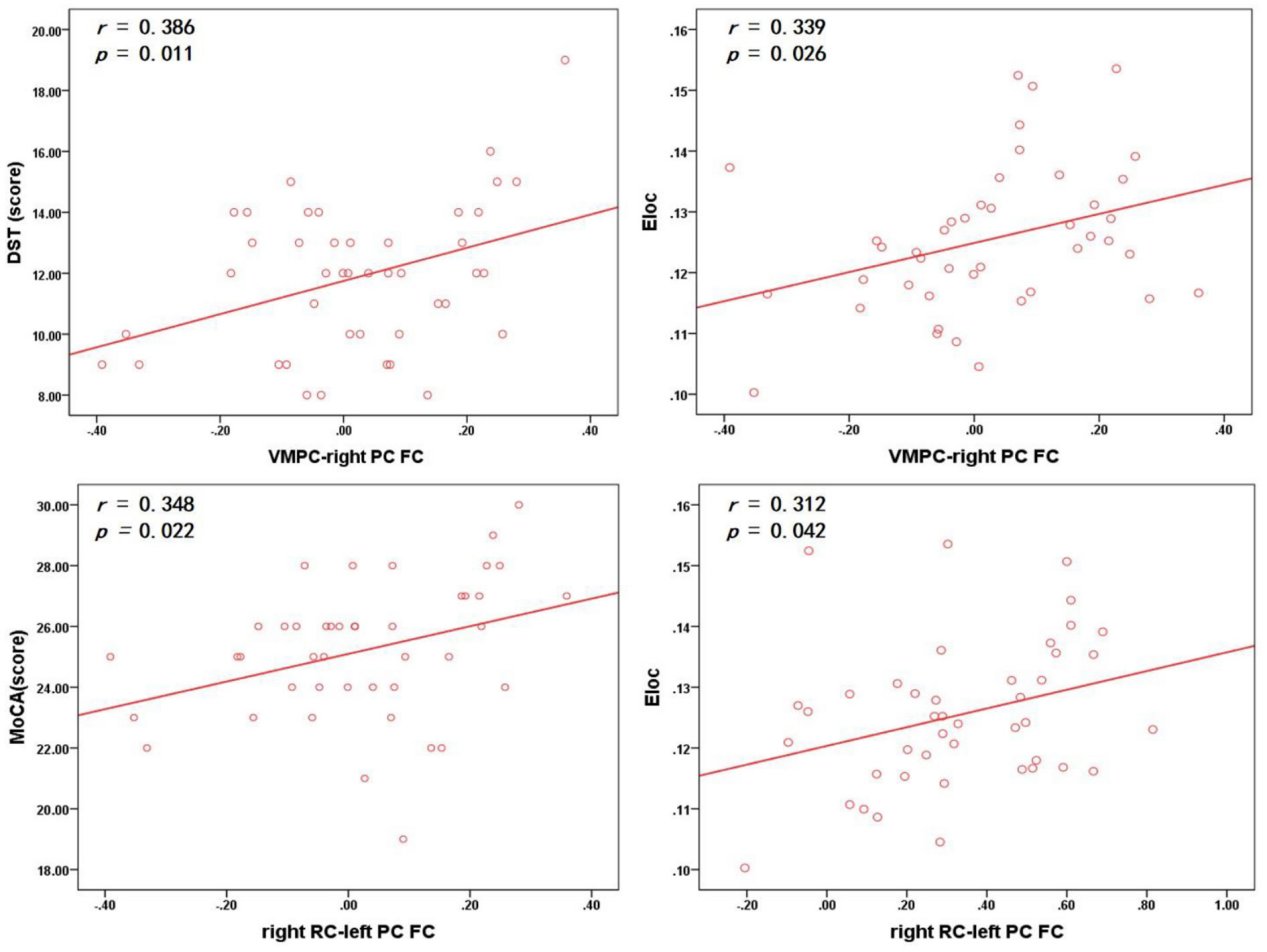
In the MHD group, the VMPC–right PC RSFC was positively correlated with the DST score and E_loc_ (uncorrected); the right RC–left PC RSFC was positively correlated with the MoCA score and E_loc_ (uncorrected). MoCA, Montreal cognitive assessment; DST, Digit span test; L, left; R, right; VMPC, Ventral medial prefrontal corte; PC, parahippocampal cortex; RC, retrosplenial cortex.

The VMPC–right PC RSFC was positively correlated with the DST score (*r* = 0.386, *p* = 0.011) and E_loc_ (*r* = 0.339, *p* = 0.026). The right RC–left PC RSFC was positively correlated with the MoCA score (*r* = 0.348 *p* = 0.022) and E_loc_ (*r* = 0.312, *p* = 0.042) in the MHD group (*p* < 0.05, uncorrected; Figure 5).

## Discussion

Using graph theory, this study explored multiple topological parameters within the DMN in ESRD patients undergoing MHD in the resting state at the global, local and edge levels. Globally, the balance between integration and separation of the DMN in the MHD group was destroyed, as evidenced by decreases in C_p_, γ, σ, and E_loal_. Locally, the MHD group showed increased nodal betweenness in the left RC compared with the HC group. At the edge level, the MHD group exhibited fewer RSFCs in the MTL subsystem (including the VMPC–left PIPL, VMPC–right PC, and right RC–left PC RSFCs) compared with the HC group. Additionally, the VMPC–right PC RSFC was associated with the DST score and E_loc_, and the right RC–left PC RSFC was associated with the MoCA score and E_loc_ in the MHD group. These results reveal abnormal topological properties within the DMN in the MHD group, thus providing novel insight into the neurobiological mechanisms of cognitive impairment in MHD patients from a connectome perspective.

### MHD-related alterations in global topology metrics

The small-world network promotes functional differentiation and integration as well as information processing, while maximizing efficiency and decreasing energy consumption to achieve the optimal balance between information separation and integration, forming the basis of cognitive functional processing (41, 42). The clustering coefficient and local efficiency are the criteria for functional separation, which can reflect local inter-connectivity or cliquishness of a network (22). In the current study, although the MHD group retained small-world networks, the C_p_, γ, σ, and E_loal_ within the DMN were diminished compared with those in the HC group, findings which are consistent with those of previous ESRD-related rs-fMRI studies that used graph theory at the whole network level (26–29). Our results indicate that the specialized processing ability for local information is reduced and the balance of regulating capacity between integration and separation is lost in a way that impairs the DMN in ESRD patients undergoing MHD (43, 44). Additionally, the lower E_loc_ is associated with reductions in VMPC–right PC and right RC–left PC RSFCs, which may indicate that the damaged topology of the DMN may be caused by hypoconnectivity within the DMN.

### MHD-related alterations in local topology metrics

The RC is an important part of the MTL subsystem within the DMN that supports a series of cognitive functions, including episodic memory, navigation, imagination, and future planning (45). Notably, the RC has been found to be impaired in the most common neurological diseases involving memory impairment (46). Nodal betweenness is designated as the fraction of the shortest paths between two nodes that passes through the area in the network and represents the influence of a region on network communication (22, 47). In this study, the MHD group showed increased nodal betweenness in left RC and a lower DST score compared with the HC group. Our finding of increased nodal centrality in the RC may reflect a compensatory effect related to the decreased E_loc_ of the DMN, which potentially relates to the impaired memory characterized by low DST scores in MHD group compared with the HC group.

### MHD-related altered edges in the MTL subsystem

The MTL subsystem consists of the VMPC, PIPL, RC, PC, and HF. For episodic memory, the activated MTL subsystem gives priority to activation and increases activities in these regions (18). Therefore, anomalous functional fluctuations in the MTL subsystem may reveal memory impairment in MHD patients, as demonstrated in the present study by the correlation between the reduction in VMPC–right PC RSFCs in the MTL subsystem and the DST score in MHD group. Intriguingly, the disconnected VMPC–left PIPL RSFCs (reported in our results) in the MTL subsystem have also been observed in Alzheimer's Disease patients with severe memory impairment (48), which indicates that the disconnection of VMPC–left PIPL RSFCs is closely related to the memory decline in MHD patients. Moreover, recent evidence shows that the RC–PC, as an important cortical network, not only supports different types of memory but also likely supports different aspects of cognition (49). In the present study, disconnection of right RC–left PC RSFCs was associated with the MoCA score in MHD patients, which indicates that the right RC–left PC disconnection has a great negative impact on cognition in MHD patients.

The VMPC is associated with a variety of emotional functions, which are damaged to some extent in many neurological diseases (50). For example, a previous fMRI study found that the decrease in VMPC–right PC RSFCs in patients with chronic schizophrenia is related to impaired emotion regulation (51). It is well-established that anxiety and depression are the most common psychological disorders in patients with ESRD (52, 53). Our findings that MHD patients exhibited anxiety and depression (as measured by the SAS and SDS scales) as well as VMPC-related disconnection (involving the VMPC–right PC and the VMPC–left PIPL RSFCs) may indicate that the ESRD-related emotional loop is disrupted.

## Limitations

Some limitations of this study should be acknowledged. First, we only studied ESRD patients undergoing MHD in the current study. Graph theory analysis of data from ESRD patients not receiving dialysis or being treated with another form of dialysis should be pursued further in the future. Second, in this study, only 20 specific DMN nodes were selected to construct the connectome. Additional research using a more detailed method for DMN subregion division is needed to determine whether different divisions may affect the results. Third, the global network measures and correlation analysis results were not corrected by multiple comparisons, meaning that this study should be considered an exploratory analysis. Fourth, the sample size of this study is quite small, and our results need to be further verified in studies with large samples. Finally, this graph theory study focused only on the DMN. Other cognitive-related networks such as the executive control network and salience network are equally important. We will apply graph theory in studies of the other networks in the future.

## Conclusion

ESRD patients undergoing MHD showed local inefficiency, abnormal nodal centrality, and hypoconnectivity within the DMN, implying that the functional differentiation and local information transmission efficiency of the DMN are diminished in ESRD. Disconnection of RSFCs in the MTL subsystem may be responsible for the discoverable topological reconfiguration in ESRD patients and may significantly affect multidomain neurocognition, including memory and emotion regulation.

## Data availability statement

The raw data supporting the conclusions of this article will be made available by the authors, upon reasonable request.

## Ethics statement

The studies involving human participants were reviewed and approved by Affiliated Zhongshan Hospital to Dalian University. The patients/participants provided their written informed consent to participate in this study.

## Author contributions

All authors listed have made a substantial, direct, and intellectual contribution to the work and approved it for publication.

## Conflict of interest

The authors declare that the research was conducted in the absence of any commercial or financial relationships that could be construed as a potential conflict of interest.

## Publisher's note

All claims expressed in this article are solely those of the authors and do not necessarily represent those of their affiliated organizations, or those of the publisher, the editors and the reviewers. Any product that may be evaluated in this article, or claim that may be made by its manufacturer, is not guaranteed or endorsed by the publisher.
