# Supplementary material for: Abnormal topological parameters in the default mode network in patients with impaired cognition undergoing maintenance hemodialysis
Source: Front Neurol. 2022 Aug 18;13:951302. doi: 10.3389/fneur.2022.951302 (PMC9433780; doi:10.3389/fneur.2022.951302)
Supplement: Supplementary file 1 [file Data_Sheet_1.docx]

**Supplementary Information**

**Materials and Methods**

*Network metrics*

In order to characterize the topological organization of the functional brain networks, we employed common graph metrics to analyze the brain binary networks at each threshold value. The graph metrics we used were as follows: clustering coefficient ($C_{p}$), shortest path length ($L_{p}$), normalized clustering coefficient (*γ*), normalized shortest path length (*λ*), small-world index (*σ*), global efficiency ($E_{glob}$) and local efficiency ($E_{loc}$),nodal degree, nodal betweenness, The details and interpretations of these network measures are described below.

*The clustering coefficient* quantifies the extent of local inter-connectivity or cliquishness of a network. For a given node *i*, $C_{i}$is defined as:

$$C_{i}=\frac{2e_{i}}{k_{i}(k_{i}-1)}$$

where $k_{i}$is the degree of node *i*, and $e_{i}$ is the number of actual existing connections between the nearest neighbors of node *i*.$C_{i}$ is a ratio of the total number of edges divided by the maximum possible number of edges between the nearest neighbors of node *i*. The mean clustering coefficient of network $C_{p}$ is the average of the clustering coefficient over all nodes in a network(Watts and Strogatz, 1998):

$$C_{P}=\frac{1}{N}C_{i}$$

*The shortest path length* reflects the mean minimal travel path between any pair of nodes(e.g., node *i* and node *j*) in the network. The shortest path length of a network is defined as:

$$L_{p}=\frac{1}{N(N-1)}\sum_{i\neq j\in G} L_{ij}$$

where *N* is the number of nodes in the network, and $L_{ij}$is defined as the shortest path length between node *i* and node *j* in the network. The shortest path length of a network quantifies the average number of connections between nodes along the shortest paths(Zhu et al., 2018).

To compute the small-world properties of the network, the values of the clustering coefficient ($C_{p}$) and shortest path length ($L_{p}$) were compared with 100 matched random networks. The random networks preserve the same number of nodes, edges and degree distribution as real networks (Maslov and Sneppen, 2002). Furthermore, the normalized clustering coefficient (*γ*) and normalized shortest path length (*λ*) were calculated as follows:

$$\gamma=\frac{C_{p}^{real}}{C_{p}^{rand}}$$

$$\lambda=\frac{L_{p}^{real}}{L_{p}^{rand}}$$

where $C_{p}^{rand}$ and $L_{p}^{rand}$ are the mean clustering coefficient and shortest path length of 100 matched random networks. A network is considered to have small-world properties if *γ*>1 and *λ*~1. Then, these two parameters were unified into a simple quantitative parameter, small-worldness (*σ*). The small-world index of a network is defined as:

$$\sigma=\frac{\gamma}{\lambda}$$

A real network is said to be small-world if *σ*> 1, and has a stronger small-world property when the value of *σ* is higher.

*The global efficiency* ($E_{glob}$) of network G measures the ability of parallel information transmission in the network (Latora and Marchiori, 2001),which is computed as follows:

$$E_{glob}(G)=\frac{1}{N(N-1)}\sum_{i\neq j\in G} \frac{1}{L_{ij}}$$

where $L_{ij}$ is the shortest path length between node *i* and node *j* in network G.

*The local efficiency* ($E_{loc}$) of network G measures how much the network is fault tolerant and shows the capability of information transfer in each subgraph when node *i* is removed (Latora and Marchiori, 2001), which is defined as:

$$E_{loc}(G)=\frac{1}{N}\sum_{i\in G} E_{glob}(G_{i})$$

where $E_{glob}$ is the global efficiency of the network, and $G_{i}$denotes the subgraph composed of the nearest neighbors of node *i*.

*The nodal degree* is defined as the number (binary graph) or the total connectivity strength (weight graph) of all connections that link to a node, reflecting the centrality of this node in the network.

*The nodal betweenness: the fraction of shortest paths from node m to node n that pass through node i* (Bullmore and Sporns, 2009), which is defined as follows:


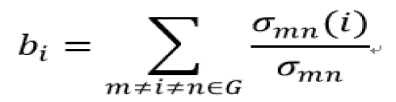


*The nodal betweenness* captures the influence of an index node over information flow between all other nodes in a network.

**References**

Latora, V., and Marchiori, M. (2001). Efficient behavior of small-world networks. *Phys. Rev. Lett.* 87, 198701. doi: 10.1103/PhysRevLett.87.198701.

Watts, D.J., and Strogatz, S.H. (1998). Collective dynamics of 'small-world' networks. *Nature* 393, 440-2. doi: 10.1038/30918.

Zhu, Y., Wang, D., Liu, Z., and Li, Y. (2018). Aberrant topographical organization in default-mode network in first-episode remitted geriatric depression: a graph-theoretical analysis. *Int. Psychogeriatr.* 30, 619-628. doi: 10.1017/S1041610218000054.

Bullmore E, Sporns O. Complex brain networks: graph theoretical analysis of structural and functional systems. Nat Rev Neurosci. 2009 Mar;10(3):186-98. doi: 10.1038/nrn2575.
